# Tracheobronchopathia Osteochondroplastica: a rare case report of a non-smoker and non-atopic patient, with a long history of wheezing since childhood

**DOI:** 10.1186/s40248-016-0050-7

**Published:** 2016-04-19

**Authors:** Alessandro G. Fois, Antonella Arcadu, Luigi Santoru, Rocco Trisolini, Vincenzo Marras, Giorgio C. Ginesu, Sara Canu, Lorenzo Cordero, Gabriella Diana, Pietro Pirina

**Affiliations:** Department of Respiratory Disease, University of Sassari, viale san Pietro 46/b, 07100 Sassari, Italy; Thoracic Endoscopy and Pulmonology Unit, Maggiore Hospital, Bologna, Italy; Pathology Unit, University of Sassari, Sassari, Italy; Department of Clinical and Experimental Medicine, Surgical Clinic, University of Sassari, Sassari, Italy

**Keywords:** Rare diseases, Respiratory sounds, Tracheal disease, Tracheobronchopathia Osteochondroplastica

## Abstract

**Background:**

Tracheobronchopathia Osteochondroplastica (TBPO) is an uncommon and benign condition characterized by osseous or metaplastic cartilaginous 1–3 mm nodules in the submucosa of the tracheo-bronchial tree. Posterior membranous wall of trachea is typically spared. Ecchondrosis and exostosis nodules can cause chronic inflammation and mucosal metaplasia, stiffness and airway obstruction. The prevalence of this disease, often asymptomatic or associated with nonspecific symptoms, is underestimated, and the mean age at diagnosis is 50 years.

**Case presentation:**

We report a case of a 49 year old male, non-smoker. He was a smith, homeless, born in Romania and reported a diagnosis of asthma since childhood. He was admitted to our Respiratory Unit presenting low-grade fever with profuse sweating, cough, purulent sputum, and ground-glass opacity with irregularity in main bronchi detected by High-Resolution Computed Tomography (HRCT) scan.

Fibrobronchoscopy revealed the presence of mucosal irregularities up to the segmental bronchi entrance. Histological examination showed nodules of osseouscartilaginous nature, consistent with TBPO. Microbiological tests of Bronchoalveolar Lavage fluid also revealed an infection by Pseudomonas Aeruginosa.

**Conclusion:**

TBPO is a rare disease characterized by wheezing, cough, hemoptysis, and recurrent pulmonary infections, with typical onset during adulthood. In the case reported, the symptoms began in childhood, although they had been misinterpreted as asthma. Even if childhood-onset is not reported in literature, it is likely that small changes occur in the first few years of life and become more evident in adulthood. The involvement of segmental and sub-segmental bronchi, usually spared in TBPO, could explain the presence of wheezing and non-productive cough reported by our patient since childhood.

## Background

Tracheobronchopathia Osteochondroplastica (TBPO) is an uncommon and benign condition affecting the trachea and, less frequently, the bronchial system. It is characterized by the presence of osseous or metaplastic cartilaginous 1–3 mm nodules in the submucosa, protruding into the tracheo-bronchial lumen. The lesions are localized in the anterolateral wall of the trachea, typically sparing the posterior membranous wall. Larger diameter nodules can cause stiffness and airway obstruction [1].

The prevalence of this disease, often asymptomatic or associated with nonspecific symptoms, is underestimated [2, 3]. An incidence of 0.01 to 4.2 per 100,000 inhabitants has been estimated, with no difference in gender distribution. The mean age at diagnosis is 50 years [2, 3].

The aetiology is currently not known. The first case of a familial occurrence of TBPO was described in 1989 [4]. In 1997 Tajima et al. hypothesized the possible involvement of a bone morphogenetic protein (Bone Morphogenetic Protein-2, BMP-2) and of TGF-beta1 (transforming growth factor-beta 1) in inducing the formation of submucosal osseous and cartilaginous nodules in the tracheobronchial system [5]. The presence of ecchondrosis and exostosis nodules in the tracheal submucosa can cause edema and thinning of the overlying mucosa, resulting in chronic inflammation and mucosa metaplasia [6].

## Case presentation

We report a case of a 49 year old male, never smoker. He was homeless, born in Romania, and had been working as a smith.

**Fig. 1:**
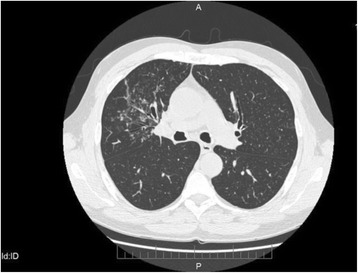
HRCT. Ground glass consolidation in anterior segment of right upper lobe

He had a clinical history of fatty liver disease (FLD) and renal lithiasis. He reported a history of wheezing since childhood, with non-productive cough, diagnosed at different stages as asthma. He had no family history of respiratory diseases, including tuberculosis (TB), or asthma and atopy. He was admitted to our Respiratory Unit with suspected pulmonary tuberculosis and with symptoms characterized by a low-grade fever with profuse sweating combined with productive cough of purulent sputum, dyspnea, wheezing, and chest pain. At diagnosis the patient showed a severe obstructive ventilatory deficit, not reversible after inhalation of short acting beta 2 agonists. A Chest X-Ray prescribed by his general practitioner showed diffuse interstitial thickening without parenchymal consolidation. He was treated with a broad-spectrum antibiotic but the symptoms persisted for a few weeks. Chest X-ray didn’t show any parenchymal consolidation, whilst a High Resolution CT scan (HRCT) showed the presence of ground-glass opacity in the anterior segment of the right upper lobe, of suspected tubercular origin (Fig. 1).

Physical examination of the chest was negative, with no superficial palpable lymphadenopathy. During hospitalization in our department of Respiratory Disease, an antibiotic therapy with ceftriaxone was established. Blood chemistry tests did not show any alteration of inflammatory indices. Sputum culture was negative for non specific flora, fungi and Mycobacterium tuberculosis. Serological tests were also negative for Mycoplasma pneumoniae, Chlamydiae, and Pneumotropic Viruses. No urinary antigen for Legionella or Pneumococcal infection was found.

However, the patient tested positive at Quantiferon-TB and Tuberculin Skin Tests.

**Fig. 2:**
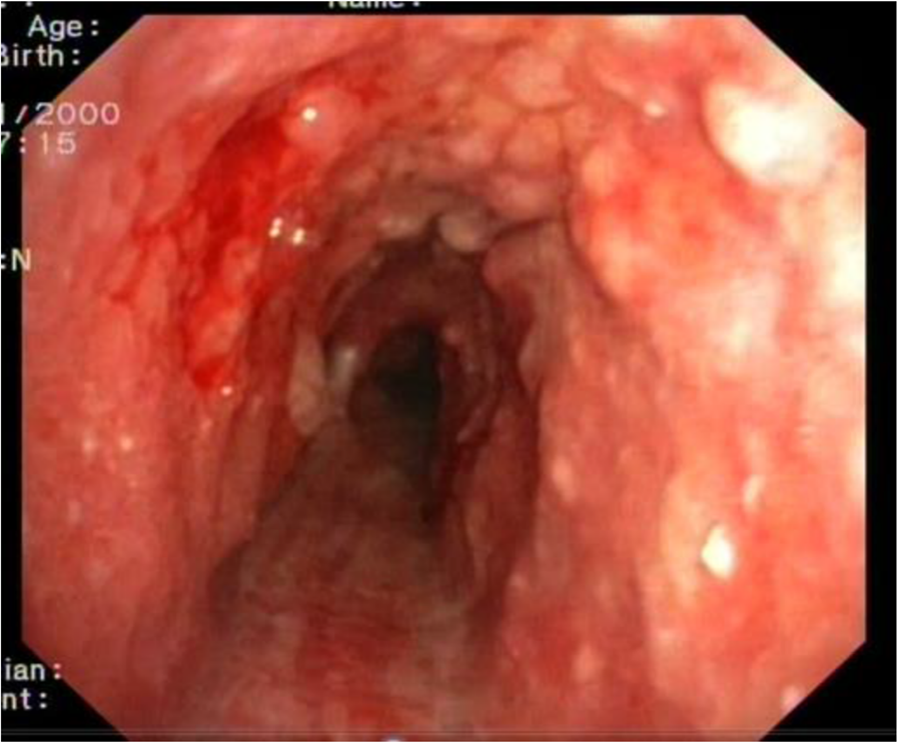
Endobronchial nodules in anterolateral wall of trachea

**Fig. 3:**
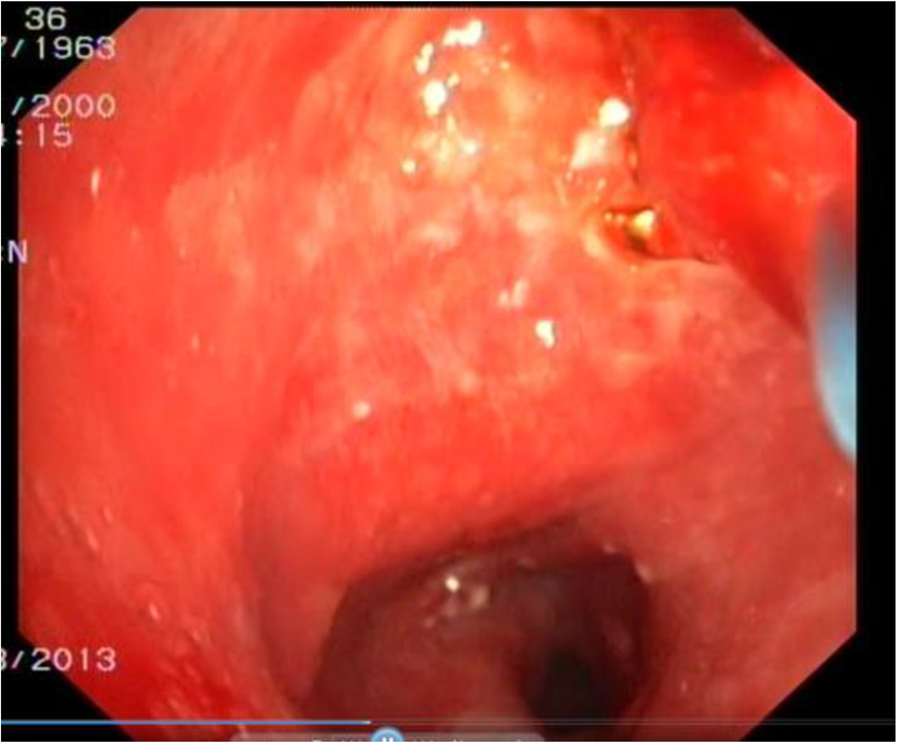
Nodules in tracheobronchial tree till the segmental bronchi entrance

A Fibrobronchoscopy finally revealed the presence of mucosal irregularities spread throughout the tracheobronchial system up to the segmental bronchi entrance, prevailing in the antero-lateral wall of the trachea and sparing the membranous pars, where a biopsy was performed (Figs. 2 and 3).

**Fig. 4:**
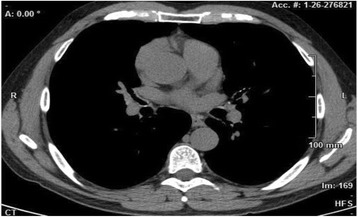
HRCT showed irregularity in main bronchi

The involvement of the segmental bronchi was also highlighted by HRCT (Fig. 4).

**Fig. 5:**
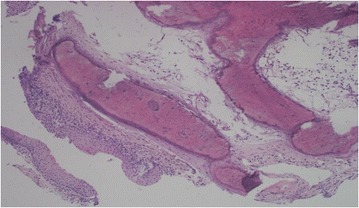
Hematoxylin-eosin 20× magnification: metaplastic bronchial epithelium with underlying nodular lesion of trabecular bone

Histological examination reported a mucosal tissue edged by a metaplastic epithelium, with underlying nodules of osseouscartilaginous nature, consistent with Tracheobronchopathia Osteochondroplastica (Fig. 5).

Microbiological tests of Bronchoalveolar Lavage fluid were negative for Mycobacterium tuberculosis and also revealed an infection by Pseudomonas Aeruginosa, with bacterial load of one-million CFU / ml. Following an antibiogram, treatment with Amikacin was established, leading to a clinical and CT scan improvement.

## Discussion

TBPO was described for the first time in 1857 by Wilks in a young man suffering from Pulmonary Tuberculosis [7]. Since then, several clinical cases have been reported, demonstrating how TBPO can be asymptomatic or associated to non-specific respiratory symptoms, such as chronic cough, dyspnoea, hemoptysis, stridor, and recurrent and slowly resolving pneumonia [8, 9]. Diagnosis is often an incidental finding at the time of bronchoscopy or during a difficult intubation [10]. The clinical examination may demonstrate the presence of rhonchi and whistles. The intensity of symptoms is correlated with the location and the extent of bronchial obstruction.

Diagnosis is usually made in adulthood. However, the onset of symptoms generally precedes the diagnosis by several years [8]. Our patient reported wheezing and non-productive cough since childhood, without the presence of typical asthmatic exacerbations. Despite the absence of atopy, the symptomatology was considered as asthma or asthma-like, but could have been probably attributed to an early onset of TBPO. We recognize that the socio-cultural status of the patient might have delayed an earlier diagnosis.

A recent retrospective cohort study of 22 patients with TBPO [2] showed that chest X-ray was positive in only 16.6 % of cases, while 81.2 % of patients had abnormalities on chest CT scan. These were characterized by irregular tracheobronchial wall thickening, calcification, and, more rarely, tracheal stenosis. Multiple submucosal nodules, with or without calcifications, sparing the posterior membranous wall, and deformation of the cartilaginous tracheal rings in absence of external compression, are pathognomonic findings at CT scan [2].

The definitive diagnosis is made by bronchoscopy, that allows to evaluate the extent and severity of the disease, showing the presence of complications such as ulcerations or tracheobronchial stenosis [2, 8]. We also want to stress the importance of bronchoscopy, not only to identify the characteristic mucosal disorders, but also to isolate the pathogens which are responsible for the recurrent infections. Our clinical case demonstrates how the TBPO can present with clinical symptoms of pneumonia and parenchymal consolidation suggestive of pulmonary tuberculosis [11, 12]. Recurrent respiratory infections and lung consolidations are common and can often drive the diagnosis in patients previously asymptomatic. In a recent study by Zhu et al. [2], recurrent respiratory infections were observed in 8 out of 22 patients (36.3 %); the same percentages have been previously reported by Nienhuis et al. [8].

In 2004 Dutau et al. [13] proposed a disease severity classification based on the extent of endoscopic lesions:

- Stage A: Scattered Nodules (few nodules with large areas of normal mucosa in between);Stage B: Diffuse Nodules (many nodules affecting the entire mucosa, without areas of normal mucosa);
- Stage B: Diffuse Nodules (many nodules affecting the entire mucosa, without areas of normal mucosa);
- Stage C: Lesions Confluent (fusion of adjacent lesions).

In Stage C confluent lesions can lead to a severe breathing impairment due to mechanical obstruction.

In unclear cases, bronchial biopsies allow the differential diagnosis of diseases such as amyloidosis, sarcoidosis, papillomatosis, tracheobronchial calcinosis and post-tuberculosis calcified lesions. These differential diagnoses should be considered, particularly in diffuse forms and in cases involving the posterior tracheal wall [14, 15]. Leske et al. [16] showed that there is no evidence of increasing incidence of malignant disease in patients with TBPO, therefore, the association of TBPO with lung cancer and Non-Hodgkin’s lymphoma [14], found in TBPO literature, should be considered as a co-incidental morbidity.

In our patient, nodules were located throughout the mucosa of tracheobronchial system, sparing the membranous trachea, and extending to the segmental and sub-segmental bronchi. In case of atypical localization of lesions (e.g. in the larynx or in the membranous trachea), it is recommended a histological examination to exclude other diagnoses as amyloidosis, sarcoidosis and papillomatosis [8, 17] .

In our case, given the medical history characterized by serotine fever, asthenia, productive cough, wheezing, Mantoux skin test and Quantiferon positivity, and CT scan findings of ground-glass opacity with tree-in-bud pattern (right upper lobe - Fig. 1), the main hypothetical diagnosis was pulmonary tuberculosis. This was also supported by the fact that the patient came from an endemic area for TB [18], and had an unhealthy lifestyle, living in random and crowded accommodations. The endoscopic specimen, however, characterized by minute and diffuse nodules in the tracheobrochial system, has allowed us to make a certain diagnosis of TBPO. Endoscopic aspects of endobronchial TB can hardly simulate those of TBPO, at least in its diffuse form [11, 12]. However, the negativity of Broncho Alveolar Lavage for Mycobacterium species and the positivity for Pseudomonas Aeruginosa allowed us to start a targeted therapy with Amikacin.

According to the endoscopic classification proposed by Dutau et al. [13], our patient would be classified as a stage B. He actually showed scattered lesions within small wide areas of normal mucosa. However, this classification does not consider the actual lesions distribution in the bronchial tree. In the case discussed here, lesions reached the bronchial segmental and sub-segmental mucosa, leading to chronic symptoms such as cough, dyspnea and wheezing, and the onset of complications such as persistent respiratory infections, pulmonary infiltrates and bronchial obstruction. This classification is therefore applicable, in our view, only in TBPO cases that involve the trachea. We think that a classification should also consider the extension of the lesions in the bronchial tree. Spirometric tests can be used to evaluate obstructive pattern in symptomatic patients with extensive disease [3]. Furthermore, during the follow up, they are helpful in assessing the deterioration of lung functions that could be observed in the most extensive diseases [8]. At diagnosis our patient showed a severe obstructive ventilatory deficit, not reversible after inhalation of short acting beta 2 agonists. The severe tracheobronchial obstruction linked to TBPO, especially if the disease involves the more peripheral bronchi (segmental and sub-segmental), can lead to respiratory failure [19, 20].

TBPO therapy includes the use of antibiotics in cases of respiratory infections which often occur in these patients. The use of inhaled corticosteroids is controversial, as it may reduce the recurrence of hemoptysis, acting on the chronic inflammation that characterizes the mucosa [2, 9]. Endoscopic and surgery treatment is reserved for cases of major bronchial obstruction and include the resection of tracheal segment, partial laryngectomy, laser removal of nodules, rigid bronchoscope dilation, and stent placement (T-Y tube) [13].

## Conclusions

TBPO is a rare disease of the tracheobronchial tree with typical onset during adulthood, characterized by wheezing, cough, hemoptysis, and recurrent pulmonary infections. In the case reported, the symptoms started in childhood, although they had been misinterpreted as asthma. Even if the childhood-onset is not reported in literature, it is likely that small changes occur in the first few years of life, and become more evident in adulthood.

Another peculiarity of our patient was the involvement of segmental and sub-segmental bronchi, usually spared in TBPO. This would explain the presence of wheezing and non-productive cough reported by the patient since childhood. The occurrence of irreversible obstructive ventilatory defect after inhalation of short acting beta 2 agonists would lead clinicians to look for other causes of wheezing.

In patients with a fixed airways obstruction which is not due to COPD, a bronchoscopy could give a significant contribution to making an effective diagnosis.

## Consent

The patient has given informed consent to publication.
